# Changing Neighborhood Income Deprivation Over Time, Moving in Childhood, and Adult Risk of Depression

**DOI:** 10.1001/jamapsychiatry.2024.1382

**Published:** 2024-07-17

**Authors:** Clive E. Sabel, Carsten Bøcker Pedersen, Sussie Antonsen, Roger T. Webb, Henriette Thisted Horsdal

**Affiliations:** 1 Department of Public Health, Aarhus University, Aarhus, Denmark; 2 Big Data Centre for Environment and Health, Aarhus University, Aarhus, Denmark; 3 Centre for Integrated Register-based Research, Aarhus University, Aarhus, Denmark; 4 School of Geography, Earth and Environmental Sciences, University of Plymouth, Plymouth, United Kingdom; 5 National Centre for Register-Based Research, Aarhus University, Denmark; 6 Hammel Neurorehabilitation Centre and University Research Clinic, Aarhus University, Hammel, Denmark; 7 Centre for Mental Health and Safety, Division of Psychology and Mental Health, University of Manchester, Manchester, United Kingdom

## Abstract

**Importance:**

Complex biological, socioeconomic, and psychological variables combine to cause mental illnesses, with mounting evidence that early-life experiences are associated with adulthood mental health.

**Objective:**

To evaluate whether changing neighborhood income deprivation and residential moves during childhood are associated with the risk of receiving a diagnosis of depression in adulthood.

**Design, Setting, and Participants:**

This cohort study included the whole population of 1 096 916 people born in Denmark from January 1, 1982, to December 31, 2003, who resided in the country during their first 15 years of life. Individuals were followed up from 15 years of age until either death, emigration, depression diagnosis, or December 31, 2018. Longitudinal data on residential location was obtained by linking all individuals to the Danish longitudinal population register. Statistical analysis was performed from June 2022 to January 2024.

**Exposures:**

Exposures included a neighborhood income deprivation index at place of residence for each year from birth to 15 years of age and a mean income deprivation index for the entire childhood (aged ≤15 years). Residential moves were considered by defining “stayers” as individuals who lived in the same data zone during their entire childhood and “movers” as those who did not.

**Main Outcomes and Measures:**

Multilevel survival analysis determined associations between neighborhood-level income deprivation and depression incidence rates after adjustment for individual factors. Results were reported as incidence rate ratios (IRRs) with 95% credible intervals (95% CrIs). The hypotheses were formulated before data collection.

**Results:**

A total of 1 096 916 individuals (563 864 male participants [51.4%]) were followed up from 15 years of age. During follow-up, 35 098 individuals (23 728 female participants [67.6%]) received a diagnosis of depression. People living in deprived areas during childhood had an increased risk of depression (IRR, 1.10 [95% CrI, 1.08-1.12]). After full individual-level adjustment, the risk was attenuated (IRR, 1.02 [95% CrI, 1.01-1.04]), indicating an increase of 2% in depression incidence for each 1-SD increase in income deprivation. Moving during childhood, independent of neighborhood deprivation status, was associated with significantly higher rates of depression in adulthood compared with not moving (IRR, 1.61 [95% CrI, 1.52-1.70] for 2 or more moves after full adjustment).

**Conclusions and Relevance:**

This study suggests that, rather than just high or changing neighborhood income deprivation trajectories in childhood being associated with adulthood depression, a settled home environment in childhood may have a protective association against depression. Policies that enable and support settled childhoods should be promoted.

## Key Points

**Question:**

Are certain neighborhood deprivation trajectories and moving during childhood associated with depression in adulthood?

**Findings:**

This cohort study found that individuals who lived in neighborhoods during childhood that were more income deprived were more likely to develop depression in adulthood. The experience of moving during childhood, whether living in a deprived or nondeprived neighborhood, was associated with significantly higher rates of depression in adulthood compared with those who did not move.

**Meaning:**

This study suggests that rather than solely high neighborhood income deprivation in childhood being associated with onset of depression during adulthood, a settled home environment in childhood may have a protective association.

## Teaser

This cohort study comprising more than 1 million individuals evaluates whether changing neighborhood income deprivation and residential moves during childhood are associated with the risk of depression in adulthood.

## Introduction

Globally, the increasing prevalence of mental health conditions poses a significant public health challenge and burden.^1^ An estimated 13% of the world’s population lived with mental health conditions in 2020.^2^ The global economic burden of mental health conditions, including reduced productivity and treatment expenses, reached approximately $2.5 trillion in 2010 and is predicted to increase to $6 trillion by 2030,^3^ exceeding the combined costs of cancer, diabetes, and chronic respiratory diseases.^2^ The causes of mental health conditions are complex and multifaceted, including biological, socioeconomic, and psychological factors; mounting evidence suggests that individuals’ natural, built, and social environments are associated with mental health.

Two key approaches informed our investigation: the exposome and the life course, both suggesting a longitudinal study design. The exposome,^4,5^ defined as the totality of an individual’s exposures over their life course, encompasses both physical (such as air pollution or green space) and social exposures. This study focuses on the social exposome, or the cumulative social exposures over the whole life course from birth onward, that influence health. The social exposome includes individual- and family-level characteristics, such as income, and neighborhood-level constructs, where the social environments in which people live and work can also affect their health positively and negatively. Social interaction within neighborhoods is an important facet of the social exposome, creating a sense of community, reciprocity, and trustworthiness—collectively known as *social capital*^6^—and of being able to control one’s surroundings, which are positively associated with mental well-being^7,8,9,10^ and may decrease the risk of depression.^11^

*Social context* refers broadly to the immediate physical and social environments in which we live, including the neighborhood built environment, culture, and individual or family socioeconomic status or deprivation. We were interested in the association of changing deprivation during childhood with health, whereby both the changing neighborhood character over time (where the individual remains static but the neighborhood character changes) and the association of an individual changing status or moving to another neighborhood are considered. Within the literature, there is robust evidence linking mental health disorders to social context.^12^ These associations include social deprivation,^13,14,15,16,17,18,19,20,21^ social disorganization,^21^ social fragmentation,^20,21^ social marginalization,^21^ social capital,^22^ residential mobility,^21,23^ income inequality,^14^ ethnic fragmentation,^13,21^ and physical illness.^24^ Childhood deprivation and adulthood depression are positively associated with each other,^25,26,27,28,29^ supporting the hypothesis of accumulation over time (ie, higher neighborhood-level income deprivation and more residential movement during childhood are associated with greater likelihood of receiving a depression diagnosis in adulthood). It is likely that deprivation in itself does not causally influence the risk of developing depression; rather, deprivation is probably 1 marker that, together with others, can be used to explain the incidence of depression.

Studies in Europe, North America, and China have found that children who move more frequently from birth until their mid-teens are more likely to subsequently experience a range of adverse outcomes, including attempted suicide, violent criminality, mental illness (mood, personality, and anxiety disorders), and substance misuse, with higher rates of both natural and unnatural deaths when followed up into middle age.^30,31,32,33,34,35^ These associations persist after accounting for major life events^36^ but are likely to be confounded by unmeasured adverse childhood experiences.^30^ In terms of mechanisms, it is postulated that residential mobility can disrupt social networks, emotional ties, family routines, and schooling. It can be especially stressful for all family members if the move is involuntary.^37^ Overall, these findings highlight the importance of considering childhood residential mobility as a potential risk factor for mental illness in later life.

To address these evidence gaps, we conducted this study using interlinked national Danish registers.^38^ We aimed to test the hypothesis that higher neighborhood-level income deprivation and more residential movement during childhood are associated with greater likelihood of receiving a depression diagnosis in adulthood.

## Methods

### Data Sources

#### Study Population

All 1 096 916 individuals born in Denmark between January 1, 1982, and December 31, 2003, and residing in Denmark during childhood (birth to 15 years of age) with both parents born in Denmark were identified through the Danish Civil Registration System.^38^ The system uses a unique personal identification number for all residents, providing data on sex, birth date, parental identification numbers, and continuously updated data on vital status and residential address. Residential addresses, geocoded at a 1-m spatial resolution, are lawfully updated within 5 days of moving, ensuring accurate and complete longitudinal records for most Danish residents. The Danish Data Protection Agency approved the study, with data access agreed on by the Danish Health Data Authority and Statistics Denmark. Data were analyzed on Statistics Denmark’s secure research platform using pseudoanonymized data. Because the study was based exclusively on registry data, informed consent from cohort members was not required in accordance with Danish legislation. This cohort study followed the Strengthening the Reporting of Observational Studies in Epidemiology (STROBE) reporting guideline. The hypotheses were formulated before data collection.

#### Depression

A total of 35 098 individuals aged 15 years or older with a discharge diagnosis of depression were identified from the Psychiatric Central Research Register.^39^ This registry has recorded all discharge diagnoses from psychiatric hospitals since 1969. Diagnoses were made using the *International Classification of Diseases, Eighth Revision* (*ICD-8*) until 1993 and the *International Statistical Classification of Diseases and Related Health Problems, Tenth Revision* (*ICD-10*) from 1994 onward for all inpatient contacts. Outpatient and emergency department contacts have been included since 1995. We identified all incident depression diagnoses (*ICD-8* codes 296.09, 296.29, 298.09, and 300.49; *ICD-10* codes F32-F33) during follow-up. These patients comprise the subset of those with more severe depression of all depression cases in Denmark,^40^ excluding those solely diagnosed in primary care settings.

#### Income Deprivation Index

An income deprivation measure was calculated for each cohort member. Details of this method appear in the eMethods in Supplement 1.

#### Covariates

Covariates included age, sex, and maternal and paternal age at cohort member’s birth in 6 age groups (12-19, 20-24, 25-29, 30-34, 35-39, and ≥40 years). We also calculated the number of residential changes (0, 1, or ≥2 moves) between 10 and 15 years of age. Linking to the Psychiatric Central Research Register,^39^ we obtained information on maternal and paternal history of mental health. These were classified hierarchically as any history of severe mental disorder (*ICD-8* codes 295.x9, 296.x9, 297.x9, 298.09, 298.19, 298.29-298.99, 299.04-299.05, 299.09, 300.49, 301.19, and 301.83; *ICD-10* codes F20-39), other mental disorder (*ICD-8* codes 290-315; *ICD-10* codes F00-F99), or no mental disorder present. For each parent, we also defined somatic illness as any comorbidity listed in the comorbidity index developed by Charlson et al^41^ (eTable 1 in Supplement 1) based on all discharge diagnoses recorded in the Danish National Patient Register.^42^ By linking to the Central Crime Register,^43^ which has registered all judicial verdicts and police decisions related to criminal charges since 1978, we defined parental imprisonment as a parent with a custodial sentence (one imposing mandatory detainment of the convicted individuals, either in prison or in some other closed therapeutic and/or educational institution). We further collected information about parental socioeconomic status from registries at Statistics Denmark.^44,45^ We defined parental income (by age, sex, and calendar year–specific quartiles on the basis of the entire population), highest educational level achieved (basic education [compulsory education for those aged 6-16 years], short or medium education [postcompulsory education before higher education degrees], or higher education), and employment status (employed, unemployed, or outside the workforce). Finally, we included information on parental death (from any cause). All covariates were defined at or shortly before the individual reached 15 years of age.

### Statistical Analysis

Statistical analysis was performed from June 2022 to January 2024. We followed up with individuals from 15 years of age until death, emigration from Denmark, depression diagnosis, or December 31, 2018, whichever came first. Data were analyzed using multilevel log-linear Poisson regression models with both individual-level and neighborhood-level variables, data zone as a random intercept,^46^ and with the logarithms of the aggregated person-years count set as an offset variable. This is equivalent to the Cox proportional hazards regression model, assuming piecewise constant incidence rates^47,48^ and allowing for a random intercept for each data zone.^49^ Multilevel survival models enable researchers to make valid inferences when examining both individual-level and neighborhood-level factors associated with disease risk.^49^ All statistical analyses were conducted using R, version 4.0.4 (R Project for Statistical Computing). The models were fitted using a Markov chain Monte Carlo method with the brm function in the brms package for R, version 2.17.0. We used 5 chains with 2500 iterations each with a burn-in of 500 iterations, leaving 10 000 iterations for estimation. Incidence rate ratios (IRRs) measure the association with outcomes of a 1-SD increase in the variable under investigation. First, we estimated the basic IRR with 95% credible intervals (95% CrIs) including only age and sex and their interaction. Next, we included all individual-level covariates in a fully adjusted model.

We defined data zones at the start of follow-up and calculated the general contextual association of data zones using the median IRR based on the formula used by Austin et al.^46^ We fitted a basic model as well as a fully adjusted model (with and without the mean income deprivation index during childhood).

We defined “stayers” as individuals who lived in the same data zone during their entire childhood (ie, each year from birth to 15 years of age) and “movers” as those who lived in 2 or more different data zones. In sensitivity analyses, we censored for either substance use disorders (*ICD-10* code F10) or schizophrenia spectrum disorders (*ICD-10* codes F20-29) to evaluate the association of comorbidities with outcomes.

## Results

A total of 1 096 916 individuals (563 864 male participants [51.4%] and 533 052 female participants [48.6%]) were followed up from 15 years of age. During follow-up, 35 098 individuals (11 370 male participants [32.4%] and 23 728 female participants [67.6%]) received a diagnosis of depression.

After full adjustment for individual-level risk factors for depression, lower parental income, employment status, and educational level were significantly associated with higher incidence of depression in adulthood (Table 1). Young maternal age and, to a lesser extent, paternal age at birth were also associated with elevated risk in adulthood. A fully adjusted IRR of 1.61 (95% CrI, 1.52-1.70) was observed for 2 or more residential moves between 10 and 15 years of age when compared with the reference group with zero moves.

**Table 1.** Individual-Level Risk Factors for Depression

Characteristic	No. of individuals with a diagnosis of depression	Incidence rate per 10 000 person-years	Incidence rate ratio (95% CrI)	
			Basic individual-level adjustment^a^	Full individual-level adjustment^b^
Residential changes (aged 10-15 y)				
0	30 636	30.7	1 [Reference]	1 [Reference]
1	3099	48.4	1.56 (1.51-1.62)	1.40 (1.35-1.46)
≥2	1363	62.5	1.99 (1.88-2.09)	1.61 (1.52-1.70)
Parental history of mental disorders, No. of parents				
0	27 929	29.1	1 [Reference]	1 [Reference]
1	3824	52.3	1.77 (1.71-1.84)	1.56 (1.50-1.61)
2	3345	66.9	2.26 (2.18-2.34)	2.00 (1.93-2.08)
Paternal age at birth, y				
12-19	350	48.0	1.46 (1.31-1.63)	1.07 (0.95-1.20)
20-24	4409	36.9	1.17 (1.13-1.21)	1.03 (1.00-1.07)
25-29	11 576	31.5	1 [Reference]	1 [Reference]
30-34	10 683	30.4	0.97 (0.94-0.99)	0.99 (0.96-1.01)
35-39	5485	33.0	1.05 (1.02-1.09)	1.03 (1.00-1.07)
≥40	2595	36.7	1.16 (1.12-1.21)	1.07 (1.02-1.12)
Maternal age at birth, y				
12-19	1166	47.7	1.55 (1.46-1.65)	1.22 (1.14-1.31)
20-24	8287	35.0	1.15 (1.12-1.18)	1.07 (1.04-1.11)
25-29	13 479	30.4	1 [Reference]	1 [Reference]
30-34	8739	31.4	1.03 (1.01-1.06)	1.02 (0.99-1.05)
35-39	2975	34.0	1.12 (1.08-1.17)	1.04 (1.00-1.09)
≥40	452	36.6	1.20 (1.09-1.32)	1.04 (0.94-1.14)
Parental Charlson comorbidities				
No	28 811	31.1	1 [Reference]	1 [Reference]
Yes	6287	40.4	1.29 (1.26-1.33)	1.16 (1.12-1.19)
Parental imprisonment				
No	32 880	31.8	1 [Reference]	1 [Reference]
Yes	2218	46.2	1.42 (1.36-1.48)	1.01 (0.96-1.05)
Parental death				
No	33 643	32.0	1 [Reference]	1 [Reference]
Yes	1455	47.4	1.46 (1.39-1.54)	0.95 (0.84-1.08)
Parental income quartile				
Q1 (lowest)	3702	42.8	1.55 (1.49-1.61)	1.22 (1.17-1.27)
Q2	7630	35.9	1.29 (1.25-1.33)	1.20 (1.17-1.24)
Q3	10 287	31.7	1.13 (1.10-1.16)	1.10 (1.07-1.13)
Q4 (highest)	12 222	28.3	1 [Reference]	1 [Reference]
Unknown	1257	47.0	1.66 (1.57-1.76)	1.03 (0.90-1.18)
Parental educational level				
Basic education	3188	44.1	1.45 (1.39-1.51)	1.09 (1.04-1.14)
Short or medium education	19 140	31.4	1.04 (1.02-1.07)	0.95 (0.93-0.98)
Higher education	10 661	30.1	1 [Reference]	1 [Reference]
Unknown	2109	44.5	1.47 (1.40-1.54)	1.04 (0.96-1.12)
Parental employment status				
Employed	31 907	31.3	1 [Reference]	1 [Reference]
Unemployed	471	49.0	1.56 (1.42-1.71)	1.08 (0.98-1.19)
Outside the workforce	1068	58.9	1.85 (1.74-1.97)	1.13 (1.05-1.20)
Unknown	1652	47.5	1.50 (1.43-1.58)	1.25 (1.10-1.41)

Abbreviations: CrI, credible interval; Q, quintile.

^a^ Adjusted for age and sex (and their interaction).

^b^ Adjusted for age and sex (and their interaction) and all other individual-level covariates (ie, residential changes [aged 10-15 years]), parental history of mental disorder, parental ages at birth, parental Charlson Comorbidity Index, parental imprisonment, parental death, parental income, parental educational level, and parental employment status.

Table 2 reports heterogeneity in the incidence rate of depression risk across data zones for the whole of Denmark: the general contextual association. This multilevel analysis reports a median IRR of 1.24 (95% CrI, 1.22-1.25) after full adjustment, which is a geographic association. This association could be real, or it could be an artifact of differential regional reporting.

**Table 2.** General and Specific Contextual Associations With Depression Risk

Depression	General contextual association across data zones, 2 competing measures		Specific contextual association of accumulated neighborhood-level deprivation during childhood, IRR (95% CrI)^a^
	Random variance (95% CrI)	Median IRR (95% CrI)^b^	
Basic individual-level adjustment^c^	0.06 (0.05-0.07)	1.26 (1.24-1.28)	1.10 (1.08-1.12)
Full individual-level adjustment^d^	0.05 (0.04-0.06)	1.24 (1.22-1.25)	1.02 (1.01-1.04)

Abbreviations: CrI, credible interval; IRR, incidence rate ratio.

^a^ The IRR measures the association of a 1-SD increase in accumulated deprivation during childhood.

^b^ The median IRR quantifies the variation between data zones (clusters) by comparing 2 identical individuals from 2 randomly chosen data zones. Consider 2 people with the same covariates chosen randomly from different data zones: the median IRR is the median between the person with the higher incidence rate and the person with the lower incidence rate.

^c^ Adjusted for age and sex (and their interaction).

^d^ Adjusted for age and sex (and their interaction), residential changes (aged 10-15 years), parental history of mental disorder, parental ages at birth, parental Charlson comorbidities, parental imprisonment, parental death, parental income, parental educational level, and parental employment status.

**Figure 1.**
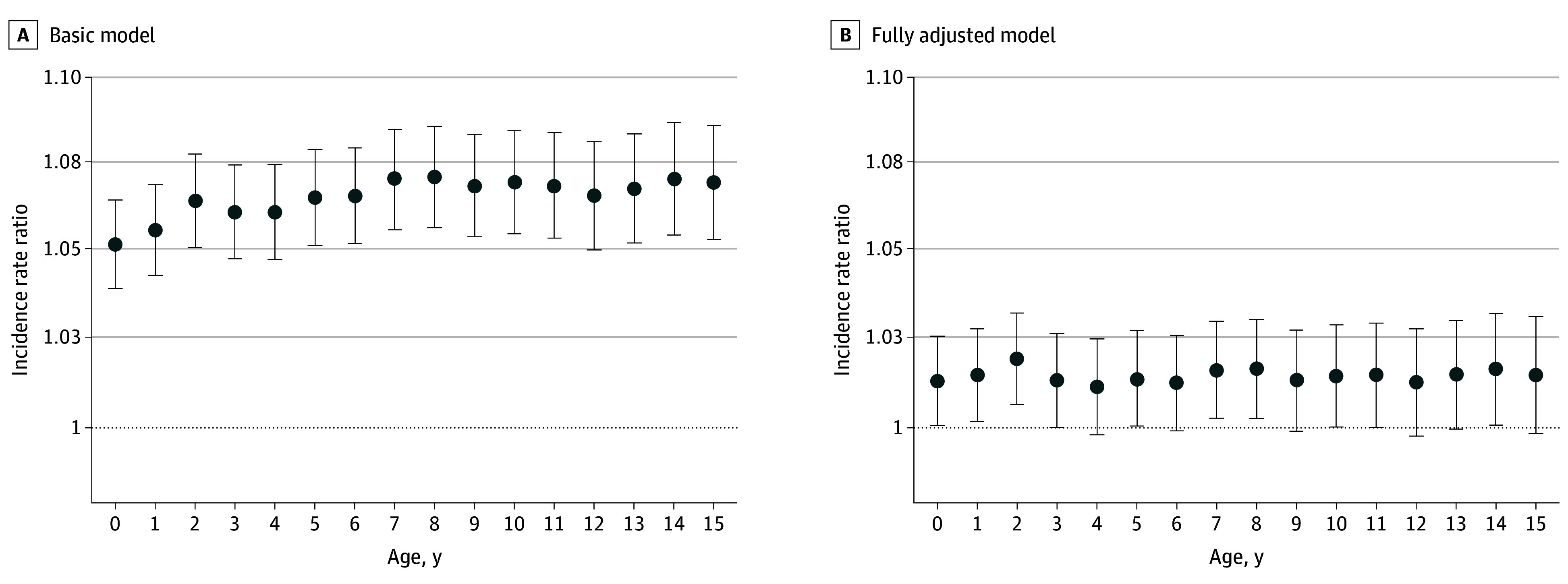
Income Deprivation Index Incidence Rate Ratios for Each Year of Childhood Incidence rate ratios measure the associations of a 1-SD increase in the income deprivation index at each age with depression in adulthood. In the basic model (A), incidence rate ratios were adjusted for age and sex (and their interaction), and in the fully adjusted model (B), incidence rate ratios were adjusted for age and sex (and their interaction), residential changes (aged 10-15 years), parental history of mental disorder, parental ages at birth, parental Charlson comorbidities, parental imprisonment, parental death, parental income, parental educational level, and parental employment status. The circles indicate the incidence rate ratios, and the error bars indicate the 95% credible intervals.

Figure 1 shows basic and full adjustment of our income deprivation index at each age from birth to 15 years of age and demonstrates a small but consistent association that greater neighborhood income deprivation at any age during upbringing is associated with increased depression risk. The IRR, representing the accumulated association of neighborhood income deprivation during childhood with risk of depression, was 1.10 (95% CrI, 1.08-1.12). After full individual-level adjustment, it was 1.02 (95% CrI, 1.01-1.04), which is higher than any individual year shown in Figure 1. This finding can be interpreted as a 1-SD increase in income deprivation exposure during the first 15 years of life and is associated with a 2% increase in depression. This finding also indicates that there is a small but significant and measurable association between neighborhood-level deprivation and depression in adulthood. Almost identical results were observed when censoring for substance use disorders or schizophrenia spectrum disorder (eTable 2 in Supplement 1).

Table 3 shows adulthood depression by change in the income deprivation index from birth to 15 years of age, disaggregated by quintiles. After full adjustment, an association with depression in adulthood was found of being born in a neighborhood with lower mean income deprivation (quintile 1 [Q1]) but residing in a neighborhood with higher mean income deprivation (Q5) at 15 years of age, when the IRR shifts from the reference 1.00 to 1.18 (95% CrI, 1.06-1.32). This finding suggests that being born in a Q1 neighborhood but residing in a Q5 neighborhood at 15 years of age is associated with 18% higher rates of depression. Conversely, being born in a Q5 neighborhood but residing in a Q1 neighborhood at 15 years of age results in an IRR of 1.13 (95% CrI, 1.02-1.22) or a 13% higher depression rate than the reference category. For this same Q5 group, no significant additional neighborhood-level association was found after adjusting for individual-level factors if the person resided in a Q5 neighborhood at both birth and 15 years of age. There are significant downward trajectories for those born in a Q1 neighborhood but moving to lower quintiles by 15 years of age. Conversely, we observed lower IRRs for those born in lower quintiles but moving to a Q1 or Q2 neighborhood by 15 years of age.

**Table 3.** Association of Change in Income Deprivation Index From Birth to 15 Years of Age With Adulthood Depression

Income deprivation at birth^a^	Income deprivation index at 15 y (95% CrI)^a^					Trend estimate (95% CrI)^b^
	Q1	Q2	Q3	Q4	Q5	
**Basic individual-level adjustment** ^c^							
Q1 (lowest)	1 [Reference]	1.11 (1.03-1.19)	1.25 (1.14-1.37)	1.31 (1.18-1.45)	1.42 (1.27-1.58)	1.45 (1.33-1.58)
Q2	1.05 (0.98-1.12)	1.07 (1.00-1.13)	1.14 (1.06-1.22)	1.18 (1.08-1.27)	1.31 (1.19-1.44)	1.24 (1.14-1.35)
Q3	1.15 (1.06-1.24)	1.07 (1.00-1.14)	1.13 (1.06-1.20)	1.14 (1.07-1.22)	1.23 (1.14-1.33)	1.11 (1.02-1.20)
Q4	1.25 (1.15-1.37)	1.16 (1.08-1.24)	1.11 (1.04-1.18)	1.13 (1.06-1.20)	1.23 (1.15-1.32)	1.01 (0.92-1.09)
Q5 (highest)	1.25 (1.16-1.34)	1.38 (1.28-1.48)	1.21 (1.13-1.30)	1.22 (1.15-1.31)	1.24 (1.16-1.32)	0.94 (0.88-1.02)
Trend estimate^c^	1.28 (1.19-1.37)	1.23 (1.14-1.32)	1.00 (0.92-1.08)	0.99 (0.91-1.07)	0.91 (0.83-0.98)	NA
**Fully individual-level adjustment** ^d^							
Q1 (lowest)	1 [Reference]	1.06 (0.98-1.13)	1.11 (1.02-1.22)	1.12 (1.01-1.24)	1.18 (1.06-1.32)	1.19 (1.09-1.30)
Q2	1.01 (0.95-1.08)	1.02 (0.96-1.08)	1.05 (0.99-1.13)	1.03 (0.94-1.11)	1.08 (0.98-1.18)	1.07 (0.98-1.16)
Q3	1.07 (0.99-1.16)	1.00 (0.94-1.07)	1.04 (0.98-1.11)	1.02 (0.95-1.09)	1.03 (0.95-1.12)	0.99 (0.91-1.08)
Q4	1.15 (1.06-1.25)	1.05 (0.98-1.13)	1.00 (0.94-1.07)	1.01 (0.95-1.08)	1.06 (0.99-1.14)	0.95 (0.87-1.03)
Q5 (highest)	1.13 (1.05-1.22)	1.17 (1.08-1.26)	1.04 (0.97-1.11)	1.06 (0.99-1.13)	1.04 (0.98-1.11)	0.90 (0.84-0.97)
Trend estimate^e^	1.15 (1.07-1.23)	1.10 (1.02-1.18)	0.93 (0.86-1.01)	0.99 (0.91-1.07)	0.93 (0.85-1.01)	NA

Abbreviations: CrI, credible interval; NA, not applicable; Q, quintile.

^a^ Disaggregated by quintiles.

^b^ For each income deprivation index at birth, the trend estimate measures the association with depression of Q5 income deprivation index at 15 years of age vs Q1 income deprivation index at 15 years of age.

^c^ Adjusted for age and sex (and their interaction).

^d^ Adjusted for age and sex (and their interaction), residential changes (aged 10-15 years), parental history of mental disorder, parental ages at birth, parental Charlson comorbidities, parental imprisonment, parental death, parental income, parental educational level, and parental employment status.

^e^ For each income deprivation index at 15 years of age, the trend estimate measures the association with depression of Q5 income deprivation index at birth vs Q1 income deprivation index at birth.

**Figure 2.**
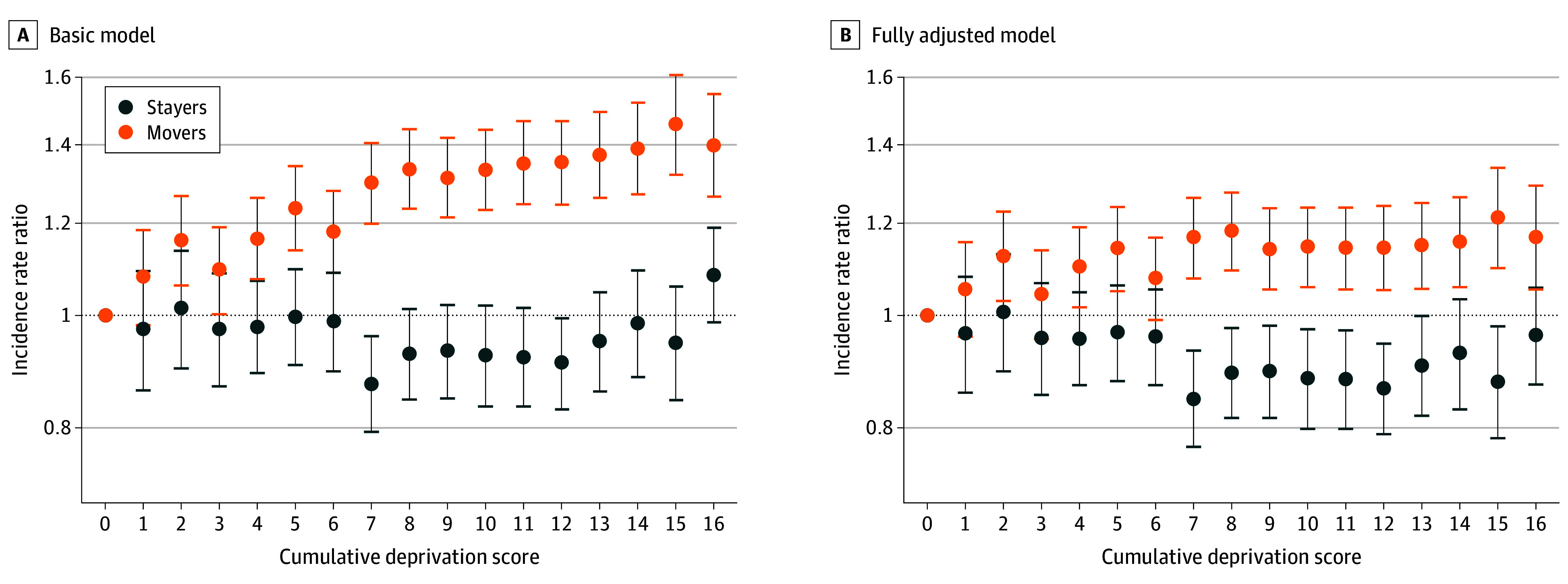
Association of Adult Depression With Cumulative Income Deprivation Score During Childhood by Stayers and Movers Incidence rate ratios measure the associations of a 1-SD increase in the income deprivation index at each score with depression in adulthood. In the basic model (A), incidence rate ratios were adjusted for age and sex (and their interaction), and in the fully adjusted model (B), incidence rate ratios were adjusted for age and sex (and their interaction), residential changes (aged 10-15 years), parental history of mental disorder, parental ages at birth, parental Charlson comorbidities, parental imprisonment, parental death, parental income, parental educational level, and parental employment status. The error bars indicate the 95% credible intervals.

Concerning the association of residential moves during childhood with adulthood depression diagnosis, we observed an IRR of 1.40 (95% CrI, 1.35-1.46) for 1 move and 1.61 (95% CrI, 1.52-1.70) for 2 or more moves after full adjustment for individual-level covariates. Figure 2 shows the cumulative income deprivation score during childhood after basic and full adjustment, comparing stayers vs movers, and it shows a marked difference between the stayer and mover groups. It appears that no matter whether living in a more- or less-deprived neighborhood during childhood, staying is potentially protective against adulthood depression.

## Discussion

To our knowledge, this is the first study to examine the association of neighborhood-level income deprivation during childhood with adulthood depression in Denmark. We have high statistical power in a complete national cohort with very low attrition. There are international studies that consider this association, but they did not capture information as richly at the individual level and a whole national cohort was not examined.^11,13,14^ Local variation or heterogeneity in depression rates between data zones across Denmark was observed, as expected.

Our analysis has shown that depression risk in adulthood is higher for individuals who lived in more deprived neighborhoods during childhood (IRR, 1.10 [95% CrI, 1.08-1.12]), which was attenuated after full individual-level adjustment (IRR, 1.02 [95% CrI, 1.01-1.04]). A 1-SD increase in income deprivation exposure was associated with a 2% increase in depression incidence after full adjustment. We did not find any specific age group during childhood that was more strongly associated with depression in adulthood, but we found that greater neighborhood income deprivation at any age during upbringing was consistently associated with increased depression risk, implying that no particular age at exposure during childhood is more strongly associated with subsequent depression risk, pointing toward an exposure accumulation hypothesis.

We observed 1.61 times the risk of developing depression in adulthood, after full adjustment, if a child moved more than once between 10 and 15 years of age. Following an earlier study,^50^ we then divided our cohort into movers and stayers. We report a key finding that the experience of moving during childhood, whether living in a deprived or nondeprived neighborhood, is associated with significantly higher rates of depression in adulthood compared with not moving after adjusting for socioeconomic status and multiple individual- and family-level confounders. Our results show that not moving during childhood is potentially protective against subsequently developing depression. Other investigators have also reported that childhood residential mobility is associated with mental illnesses in Denmark but for different time periods, and those studies did not explicitly explore neighborhood deprivation.^32,34^ We postulate that it is not the move, per se, but rather the change of neighborhood that is disruptive. The association of the distance of the move with schizophrenia risk has been investigated, with local changes of address evidently having a negligible association compared with intermunicipality moves.^51^ A fixed place of residence during childhood could be an indicator of a stable family or that individuals and families have stronger roots in their neighborhoods. These are the aspects of social support systems (such as trust and exchange of favors from neighbors, schools, and social, sporting, and religious organizations) that create a feeling of belonging and being connected, collectively known as social capital.^6^ In terms of policy implications, our findings add to the international evidence suggesting designing urban neighborhoods that support positive mental health, which specifically provide opportunities to enhance social structure in local neighborhoods.

There are many potential reasons why moving in childhood might be associated with a later likelihood of developing depression. Children of a family that is unstable, perhaps with relationship breakups or loss of employment, are more likely to need to move. The move itself could sever social ties and contribute to the breakdown of informal and formal social support services, including schooling. We acknowledge that the wider social determinants of health are highly complex, and we do not have full information about the childhood life events of our participants to establish a causal pathway, but the importance and complexity of potential child-parent trajectories after separation and the association with mental health have been illustrated elsewhere.^52,53,54^

### Limitations

This study had several limitations. First, the cases were identified from the Psychiatric Central Research Register, in which diagnoses are made in specialist secondary care settings, thereby representing the more severe end of the depression spectrum. This is a relatively small subset of all diagnosed cases of depression, most of which are made in primary care by general practitioners.^40^ Conversely, our stringent criteria likely ensure diagnostic accuracy. We would speculate that the findings would hold, albeit with a weaker observed association, if individuals who received a diagnosis in primary care were included. Second, after adjusting from basic- to full-level associations in the analysis, the apparent influence of neighborhood remains, but the association is attenuated. Thus, there might be some degree of undetected residual confounding due to imperfectly characterizing neighborhoods, imperfect measurement of covariates, or because key potential confounders were not included. Third, the definition of moving in the Danish registers, while better than in most studies, cannot fully capture the complexity of blended families. We are cognizant that, for example, in a situation where a family breakup leads to 2 parental homes, the Danish registration system will only record 1 address per child and not fully record the true lived experience of the children affected, where they might alternate between homes on a weekly or biweekly basis.

## Conclusions

This study suggests that rather than solely low neighborhood income deprivation in childhood being associated with onset of depression during adulthood, a settled home environment in childhood may have a protective association. Future research can examine whether alternative deprivation measures, such as educational or composite indices, function similarly to our income deprivation index. It will be crucial to uncover causal mechanisms between childhood experiences at the individual and neighborhood levels and adult depression. To explore neighborhood social connections, using voting participation rates as a proxy for social capital is an option. A further question worth investigating is whether the distance of a move during childhood makes a difference in adult depression. Is a move to the next road the same as moving across the country? Conceptually, moving beyond the local neighborhood, possibly changing schools, should disrupt social capital more than a local move. Or does transitioning between urban and rural areas warrant further exploration?

## Notes

Supplement 1.
eMethods.
**eTable 1.** Diseases Included in the Charlson Comorbidity Index **eTable 2.** General and Specific Contextual Effects on Depression Risk, Censoring for Substance Use Disorders and Schizophrenia
eReferences.

Supplement 2.
Data Sharing Statement
