# Supplementary material for: Changing Neighborhood Income Deprivation Over Time, Moving in Childhood, and Adult Risk of Depression
Source: JAMA Psychiatry. 2024 Jul 17;81(9):919–27. doi: 10.1001/jamapsychiatry.2024.1382 (PMC11255978; doi:10.1001/jamapsychiatry.2024.1382)
Supplement: Supplement 1. — eMethods. eTable 1. Diseases Included in the Charlson Comorbidity Index eTable 2. General and Specific Contextual Effects on Depression Risk, Censoring for Substance Use Disorders and Schizophrenia eReferences. [file jamapsychiatry-e241382-s001.pdf]

Supplementary Online Content

Sabel CE, Pedersen CB, Antonsen S, Webb RT, Horsdal HT. Changing neighborhood income deprivation over time, moving in childhood, and adult risk of depression. *JAMA Psychiatry.* Published online July 17, 2024. doi:10.1001/jamapsychiatry.2024.1382

eMethods.
eTable 1. Diseases Included in the Charlson Comorbidity Index
eTable 2. General and Specific Contextual Effects on Depression Risk, Censoring for Substance Use Disorders and Schizophrenia
eReferences.

This supplementary material has been provided by the authors to give readers additional information about their work.

© 2024 Sabel CE et al. *JAMA Psychiatry.*

eMethods.

Income Deprivation Index

Due largely to the availability of the individual-level registers in Denmark, and the lack of a decadal national census, there is no standardised deprivation index in Denmark (cf. Carstairs, Jarman and IMD indices in the UK) [1]. There is also no consistent and homogeneously sized small-area geographic delineation available in Denmark. Thus, Danish studies investigating neighborhood-level influences on health have been sparse.

In an earlier paper, we used geographic zone design theory to create a new national homogenous small-area delineation of 1,885 neighborhoods, or ‘Data Zones’ with a mean population of 2500 [2]. We aggregated individual income measures to create ‘Data Zones’ level indices of neighborhood income. Income measures were derived from the registers at Statistics Denmark, [3] which are complete from 1981 onwards. We calculated the annual proportion of individuals aged 25-64 years with gross income in the first (lowest) and second octile in each data zone. Octiles were defined based on the entire income distribution among all individuals aged 25-64 years in each year. Further, we defined the annual mean gross income among individuals aged 25-64 years in each data zone. Individuals with missing information were excluded from all calculations. To reduce uncertainty, we averaged the three income measures in 5-year bands (1981-1984, 1985-1989, 1990-1994, 1995-1999, 2000-2004, 2005-2009, 2010-2014, 2015-2018), and standardized them. We performed exploratory factor analysis on the standardized averaged income measures for each 5-year band, using maximum-likelihood methods, retaining one factor. Then the resulting factor score, representing the income deprivation index, was further standardized, and divided into quintiles with the lowest quintile representing the least deprived. The income deprivation index at residence during childhood was defined as the factor in the prior year band, e.g. the income deprivation index at birth for a person born in 2000 is the standardized factor from 1995-1999. We defined the income deprivation index at residence at each year from birth to age 15, and calculated a mean income deprivation index for the entire childhood (i.e. from birth to 15th birthday). Finally, based on the quintiles, we defined a cumulative income deprivation score summing quintiles at age 0, 5, 10, and 15. This score ranges from 0 to 16, with a value of 0 indicating low income deprivation in all four ages, and 16 representing the highest income deprivation in each of the four ages.

© 2024 Sabel CE et al. *JAMA Psychiatry*.

eTable 1. Diseases Included in the Charlson Comorbidity Index

Disease	ICD-8	ICD-10
Myocardial infarction	410	I21, I22, I23
Congestive heart failure	427.09, 421.10, 427.11, 427.19, 428.99, 782.49	I50, I11.0, I13.0, I13.2
Peripheral vascular disease	440, 441, 442, 443, 444, 445	I70, I71, I72, I73, I74, I77
Cerebrovascular disease	430-438	I60-I69, G45, G46
Dementia	290.09-290.19, 293.09	F00-F03, F05.1, G30
Chronic pulmonary disease	490-493, 515-518	J40-J47, J60-J67, J68.4, J70.1, J70.3, J84.1, J92.0, J96.1, J98.2, J98.3
Connective tissue disease	712, 716, 734, 446, 135.99	M05, M06, M08, M09, M30, M31, M32, M33, M34, M35, M36, D86
Ulcer disease	530.91, 530.98, 531-534	K22.1, K25-K28
Mild liver disease	571, 573.01, 573.04	B18, K70.0-K70.3, K70.9, K71, K73, K74, K76.0
Diabetes (type 1 and type 2)	249.00, 249.06, 249.07, 249.09, 250.00, 250.06, 250.07, 250.09	E10.0, E10.1, E10.9, E11.0, E11.1, E11.9
Hemiplegia	344	G81, G82
Moderate to severe renal disease	403, 404, 580-584, 590.09, 593.19, 753.10-753.19, 792	I12, I13, N00-N05, N07, N11, N14, N17-N19, Q61
Diabetes with end-organ damage	249.01-249.05, 249.08, 250.01-250.05, 250.08	E10.2-E10.8, E11.2-E11.8
Any tumor	140-194	C00-C75
Leukemia	204-207	C91-C95
Lymphoma	200-203, 275.59	C81-C85, C88, C90, C96
Moderate to severe liver disease	070.00, 070.02, 070.04, 070.06, 070.08, 573.00, 456.00-456.09	B15.0, B16.0, B16.2, B19.0, K70.4, K72, K76.6, I85
Metastatic solid tumor	195-198, 199	C76-C80
AIDS	079.83	B21-B24

© 2024 Sabel CE et al. *JAMA Psychiatry*.

eTable 2. General and Specific Contextual Effects on Depression Risk, Censoring for Substance Use Disorders and Schizophrenia

	General contextual effect across data zones, two competing measures		Specific contextual effect – accumulated neighborhood level deprivation during childhood ***
	Random Variance	Median Incidence Rate Ratio ****	Incidence Rate Ratio
**Depression without substance use disorders**			
- Basic individual-level adjustment *	0.06 (0.05 - 0.06)	1.25 (1.23 - 1.27)	1.10 (1.08-1.11)
- Full individual-level adjustment **	0.05 (0.04 - 0.06)	1.24 (1.22 - 1.26)	1.02 (1.01-1.04)
**Depression without schizophrenia spectrum disorders**			
- Basic individual-level adjustment*	0.06 (0.05 - 0.06)	1.25 (1.24 - 1.27)	1.10 (1.08 - 1.11)
- Full individual-level adjustment**	0.05 (0.04 - 0.06)	1.24 (1.22 - 1.26)	1.02 (1.00 - 1.04)

*Adjusted for age and gender (and their interaction)

**Adjusted for age and gender (and their interaction), residential changes (age 10-15), parental history of mental disorder, parental ages at birth, parental Charlson Index comorbidity, parental imprisonment, parental death, and parental income, parental education, and parental employment status.

*** The IRR measures the effect of a one-standard deviation increase in accumulated deprivation during childhood.

**** The Median Incidence Rate Ratio (MRR) quantifies the variation between data zones (clusters) by comparing two identical individuals from two randomly chosen data zones. Consider two people with the same covariates chosen randomly from different data zones, the Median Incidence Rate Ratio is the median incidence rate ratio between the person of higher incidence rate and the person of lower incidence rate.

© 2024 Sabel CE et al. *JAMA Psychiatry*.

eReferences.

1. McLennan D, Noble M, Plunkett E, et al. English indices of deprivation 2019: Technical report 2019.
2. Pedersen CB, Antonsen S, Timmermann A, et al. Urban-Rural Differences in Schizophrenia Risk: Multilevel Survival Analyses of Individual- and Neighborhood-Level Indicators, Urbanicity and Population Density in a Danish National Cohort Study. Schizophrenia Bulletin Open. 2022;3(1).
3. Petersson F, Baadsgaard M, Thygesen LC. Danish registers on personal labour market affiliation. Scandinavian Journal Public Health. 2011;39:95-98.

© 2024 Sabel CE et al. *JAMA Psychiatry*.
