# Supplementary material for: Changing Neighborhood Income Deprivation Over Time, Moving in Childhood, and Adult Risk of Depression
Source: JAMA Psychiatry. 2024 Jul 17;81(9):919–27. doi: 10.1001/jamapsychiatry.2024.1382 (PMC11255978; doi:10.1001/jamapsychiatry.2024.1382)
Supplement: Supplement 2. — Data Sharing Statement [file jamapsychiatry-e241382-s002.pdf]

Data Sharing Statement

Sabel. Changing Neighborhood Income Deprivation Over Time, Moving in Childhood, and Adult Risk of Depression. *JAMA Psychiatry*. Published June 18, 2024. doi:10.1001/jamapsychiatry.2024.1382

Data
Data available: No

Additional Information
Explanation for why data not available: Our individual level patient data is highly confidential. It is licensed from the Danish data protection agency for our use only. Others can request access to the relevant authorities for access.
